# Computer Games on the Development of Physical Motor Skills and Social Interaction in Preschool Children: Quasi-Experimental Study

**DOI:** 10.2196/33138

**Published:** 2026-08-07

**Authors:** Ing-Chau Chang, Chin-En Yen

**Affiliations:** 1 Department of Computer Science and Information Engineering, National Changhua University of Education, Changhua, Taiwan; 2 Department of Early Childhood Development and Education, Chaoyang University of Technology, 168, Jifeng E. Rd., Wufeng District, Taichung, 41349, Taiwan, 886 4-23323000 ext 4706

**Keywords:** preschool children, computer games, social interaction, motor development, preschools

## Abstract

**Background:**

As information technology becomes increasingly pervasive in early childhood education, the impact of digital gameplay on developmental outcomes has emerged as a critical research frontier.

**Objective:**

This study evaluates the efficacy of computer game–based instructional activities in enhancing the gross motor skills and social interactions of preschool children.

**Methods:**

This prospective quasi-experimental study involved 61 preschool children (aged 4‐6 y; 36 males, 25 females) recruited via convenience sampling from a private preschool in central Taiwan. The selection criteria included regular attendance and the absence of severe physical or cognitive disabilities. Participants were assigned to either an experimental group (n=41) or a control group (n=20) based on existing classroom structures. The intervention spanned 4 weeks, featuring 1-hour weekly computer game sessions integrated into the curriculum, while the control group followed the standard curriculum. Data were gathered through preintervention and postintervention assessments using standardized scales for physical motor and social interaction development, supplemented by a learning interest questionnaire and structured qualitative observations.

**Results:**

We used an analysis of covariance to assess the impact of interventions on the physical motor and social development of preschool children, adjusting for baseline differences using prior test scores as covariates. The experimental group exhibited significantly greater improvements in total physical motor development scores compared to the control group (mean 4.33, SD 0.06 vs mean 4.02, SD 0.09; *P*<.001). Specifically, marked gains were observed in advanced motor skills: standing on one leg for 5 seconds, walking heel-to-toe for 5 steps, and hopping on one leg 5 times. Post intervention, no statistically significant differences were found between groups regarding total social interaction scores. However, the children reported high engagement, with a mean preference score of 4.18 (SD 0.79). Qualitative observations of 3 children with developmental delays revealed a transformative shift from passive observation to active social engagement and positive affect.

**Conclusions:**

This study demonstrates that digital game interventions can effectively facilitate gross physical motor gains in early childhood. It is innovative in its integration of digital gameplay specifically targeted at gross motor skill acquisition. Furthermore, it differs from existing literature by providing empirical evidence of how game-based instruction can be seamlessly embedded into preschool physical education. This study highlights the potential of computer game–based instruction as a viable supplementary tool for physical education.

## Introduction

With technological advancements, information and communication technology devices such as computers have not only permeated school classrooms but have also integrated into the daily lives of young children. Computer games combine images and sound effects, effectively attracting children’s attention by presenting vivid game scenes [1]. Given that computer games provide children with numerous novel sensations, discoveries, and experiences, yet may also exert negative influences, a growing number of researchers have conducted extensive theoretical and empirical investigations into the impact of computer games on children and adolescents [1].

The primary appeal of computer games for children lies in their capacity to satisfy innate play-based instincts through structured digital engagement. Previous research involving young children in China suggests that this demographic exhibits a marked preference for activities that are physically engaging, straightforward, and highly entertaining [1]. Evidence indicates that through repetitive operation and practice, such as successfully navigating game levels or coordinating motor actions to hit targets, children derive a significant sense of perceived competence and self-efficacy. This feeling of achievement is rooted in successful goal attainment and the autonomous control they exert over the digital environment, which provides immediate reinforcement of their psychological and motor-related competence [1,2]. Furthermore, a study conducted with school-age children [2] demonstrated that narrative structures and role-playing elements within computer games fulfill requirements for social and interpersonal interaction. Consequently, games have been established as an effective health-promoting modality to foster specific health-related habits, such as improved oral hygiene and cooperative social interaction [2].

Study indicates that a majority of children worldwide gain exposure to computer games before the age of 6 years, with some even dedicating more than 1 hour daily to these activities [3]. Against this backdrop, computer games have increasingly been recognized and accepted by the academic community as a contemporary and legitimate form of play [4].

Research demonstrates that serious games exert a positive influence on the education of children from the preschool stage through primary school. By integrating educational content with entertainment elements, these games offer an engaging and highly motivational pedagogical approach [5] that enhances learning outcomes and academic performance [6]. Notably, the interactive nature of serious games, often requiring precise manipulation or gesture-based input, supports the development of fine motor skills and sensorimotor coordination, which are foundational for early childhood development [7]. Additionally, they enhance students’ learning motivation, aiding them in maintaining concentration and focus on specific activities [8]. Consequently, this increases learning and teaching satisfaction and creates an interactive pedagogical environment [9] that facilitates dynamic communication between instructors and learners.

Early childhood represents a critical period for physical motor development [10]. The acquisition of fundamental motor skills not only influences children’s current participation in physical activities but also carries profound implications for long-term health promotion [11]. Research has indicated that computer game interventions can effectively enhance the motor abilities of young children [5]. For instance, a study by Vernadakis et al [12] demonstrated that active video game–based intervention programs exert a positive influence on children’s fundamental motor skills. This suggests that the interactive nature of such games can foster intrinsic exercise motivation by satisfying the psychological needs for enjoyment and perceived competence during physical activity [12]. When children feel capable and find the activity inherently rewarding, their persistence in physical tasks increases. Gao et al [13] demonstrated that video games not only significantly improve preschoolers’ motor skills but also bolster their subjective evaluation of physical abilities, which, in turn, mediates the increase in physical activity. While virtual reality task-oriented training has also shown promise in improving upper extremity motor function [14], it is important to note that divergent findings exist. Some studies [15,16] report no significant impact on fundamental motor skills, suggesting that the efficacy of digital play may depend on the specific design and pedagogical integration of the game.

Social development in early childhood is fundamentally based on social and emotional learning, which consists of 5 core components: self-awareness, self-management, social awareness, relationship skills, and responsible decision-making [17]. From a scientific perspective, these social skills are not just emotional responses; they are deeply connected to cognition. For example, when a child regulates their emotions during a game, they are using cognitive skills to evaluate social cues and decide on the best way to react. Robust social development is highly correlated with a child’s future interpersonal interactions, mental health, and cognitive abilities [3]. However, the rising trend of social isolation and loneliness among modern children has elicited concerns among educators regarding whether teacher-centered, lecture-based pedagogical models can effectively foster these core competencies [18]. The role of computer games in this process is a key topic of discussion. Many researchers argue that collaborative gameplay provides a practical environment for teamwork and communication [3]. Studies show that children engage in important social behaviors during play, such as collective discussion and mutual problem-solving, which require them to use both logical thinking and emotional understanding at the same time [4]. On the other hand, without proper guidance, excessive gaming in isolation may lead to social withdrawal or a decrease in verbal communication skills [3,4].

In recent years, the population of students with special educational needs has shown an increasing trend, with computer game–based instructional interventions as a focal point of research within the field of special education. Computer games provide a safe, predictable environment characterized by low social pressure, which allows young children to engage in the repetitive practice of social skills within a scaffolded learning framework [19]. From a social cognitive perspective, these platforms reduce the cognitive load associated with complex face-to-face interactions, enabling children to focus on acquiring specific social cues. Research concerning children with intellectual disabilities has demonstrated that gamified learning can effectively overcome the challenge of low intrinsic learning motivation. By fostering a sense of autonomy and providing immediate feedback, these tools enhance students’ internal drive and self-efficacy, thereby improving their performance across conceptual, social, and practical skill domains [20]. For young children with intellectual disabilities, computer games reinforce participation motivation through incentive mechanisms such as badges and points. Compared to teacher-led direct instruction and static paper-and-pencil exercises, games transform complex social scenarios into operable, concrete tasks. Unlike traditional classroom-based rote learning, digital games provide immediate feedback and multisensory stimulation, which in turn improves children’s social adaptability in daily life [20]. Furthermore, studies indicate that children with autism are generally receptive to computer-based interventions and exhibit significant learning progress through training across various technologies, including computer-assisted instruction, electronic games, and virtual environments [21,22]. Research investigating the use of serious games in instructional activities for children with autism has found significant efficacy in enhancing core deficits, including communication, joint attention, and reciprocal social behavior. Improvements are particularly pronounced in the domain of adaptive social functioning; thus, serious games serve as a valuable tool for educators working with children on the autism spectrum by providing structured, repeatable social scenarios that build social self-efficacy [19,23].

Digital play has become an integral component of early childhood development, shifting the landscape of peer interaction. Recent empirical evidence suggests that digital game–based environments can facilitate prosocial behaviors, such as sharing and turn-taking [24]. This process is driven by a synergistic relationship among motor skills, motivation, and social competence. In the context of digital play, motor skills (eg, fine motor coordination and interface navigation) serve as the functional foundation; proficiency in these skills reduces the cognitive load required for gameplay, thereby freeing cognitive resources for navigating complex social interactions. This engagement is sustained by intrinsic motivation, which is amplified by the immediate feedback loops inherent in digital platforms. Such motivation fosters behavioral persistence, encouraging children to remain engaged in coplaying scenarios even when encountering social friction. Consequently, digital coplaying provides a scaffolded environment where children can simultaneously refine their motor coordination and practice conflict resolution [24].

To address these theoretical intersections, the objective of this study is to evaluate the impact of a computer game–based intervention on the physical motor and social interaction development of preschool children. Specifically, the study tests the following hypotheses:

- Hypothesis 1: Children participating in the computer game intervention (experimental group) will demonstrate significantly greater gains in gross motor skills compared to those in the standard curriculum (control group).
- Hypothesis 2: The digital intervention will bolster children’s intrinsic learning motivation and engagement levels.
- Hypothesis 3: Digital game environments will foster improved social interaction development, enabling children to resolve interpersonal conflicts more effectively.

## Methods

### Study Design

This study used a 2×2 quasi-experimental mixed design to evaluate the effectiveness of the intervention. The design involved 2 primary factors: group (experimental group vs control group) as the between-participants variable and time (pretest vs posttest) as the within-participants variable.

A quasi-experimental approach was selected because participants were assigned to groups based on their preexisting intact classes rather than through individual random assignment. This method was chosen to minimize disruption to the participants’ natural learning environment.

The experimental group received a computer game–based intervention once a week (1 hour per session) for 4 consecutive weeks. Conversely, the control group did not participate in any computer game instruction and continued with their regular curriculum and daily routines. Assessment of motor skills and social interaction was conducted for both groups at 2 time points: prior to the intervention (pretest) and immediately following the 4-week period (posttest).

### Participants Recruitment

The study was conducted at a private preschool in central Taiwan, an institution comprising 4 individual classes. A convenience sampling strategy was used to recruit participants from the middle and senior classes (children aged 4 to 6 y). To minimize instructional disruption within the school setting, a cluster assignment approach was used: two classes were assigned to the experimental group (n=41), and 1 class served as the control group (n=20). The experimental group consisted of 25 males (25/41, 60.9%) and 16 females (16/41, 39.0%), while the control group included 11 males (11/20, 55%) and 9 females (9/20, 45%). The experimental group participated in a 4-week computer game–based instructional intervention, whereas the control group followed the preschool’s standard curriculum. The selection of this sample was guided by the principle of developmental appropriateness. Based on prior pedagogical assessments, the game interface usability and instructional design were meticulously aligned with the cognitive levels of preschool children in the middle and senior classes, ensuring that all participants could navigate and operate the system effectively.

### Assessment Instrument

#### Demographic Data of Participants

Personal background information of the participating children was collected, encompassing variables such as name, gender, age, height, weight, and birth order. Additionally, the educational attainment and occupation of the primary caregivers were recorded. This information was provided directly by the primary caregivers to ensure the accuracy and completeness of the demographic profile.

#### Physical Motor Development Assessment Scale

The physical motor development assessment scale for preschool children used the “Localized Standardized Developmental Screening Tool for Children Aged 4‐6,” promulgated by the Ministry of Health and Welfare of Taiwan. This tool is a validated diagnostic instrument widely used in clinical and educational settings across Taiwan for developmental monitoring. Since this is a prevalidated standardized instrument with established psychometric properties, its structural validity is inherently supported by national norms. Classroom teachers evaluated the children based on their daily physical and motor performance. The assessments were conducted before and after the computer game intervention, respectively. This scale consists of 8 items (eg, can stand on one leg steadily for at least 3 s) to evaluate motor coordination, balance, lower limb control, and motor stability. Classroom teachers evaluated children on a 4-point Likert scale. Points from 1 to 4 were assigned based on the frequency of behavioral occurrences (never, occasionally, frequently, and always). Higher scores indicate a higher frequency of the specific behavior and reflect superior performance in the child’s development. Regarding reliability, the scale demonstrated high internal consistency, with a Cronbach α of 0.95 before the test and 0.94 after the test.

#### Social Interaction Development Assessment Scale

The social interaction development assessment scale for preschool children used the “Localized Standardized Developmental Screening Tool for Children Aged 4‐6,” promulgated by the Ministry of Health and Welfare of Taiwan. This scale, as a standardized assessment tool, possesses high content validity and expert-vetted structural integrity. Classroom teachers evaluated the children based on their daily social behavioral performance. Assessments of social behavioral development were conducted before and after the computer game intervention, respectively. It comprises 5 items (eg, plays games with others with varied themes and styles) covering dimensions such as peer cooperation, turn-taking, adherence to rules, and emotion regulation. It used a 4-point Likert scale for scoring, with points from 1 to 4 assigned based on the frequency of behavioral occurrences (never, occasionally, frequently, and always). The social development assessment scale contains reverse-scored items (eg, lack of patience for waiting), for which the scoring was converted accordingly. Higher scores represent superior developmental performance in the respective social dimensions. The instrument showed strong reliability, with Cronbach α values of 0.8 and 0.9 for pretests and posttests, respectively.

#### Learning Interest Questionnaire

To evaluate the experimental group’s overall interest in the game-based learning activities, a single-item interest scale was administered post intervention. This measure aimed to capture the children’s affective attitudes toward the computer games. Given the cognitive developmental stage of preschool children, a simplified 5-point Likert scale was used, with visual aids (eg, smiley faces) ranging from “dislike very much” (1) to “like very much” (5).

#### Observation Record

The researchers observed and documented the children’s behavioral responses and emotional states in situ within the instructional environment. This qualitative data was used to evaluate the efficacy of the computer game intervention.

#### Computer Games

This study used the Scratch (Lifelong Kindergarten Group at the MIT Media Lab) visual block–based programming software to develop original computer games, using its graphical interface for intuitive game creation. Video sensing technology was leveraged to facilitate real-time interaction between physical movements and digital scenarios. By integrating young children’s daily life experiences with their developmental requirements, the research created engaging learning environments through vibrant characters and lively animated scenarios. Furthermore, through the provision of visual and auditory stimuli alongside operational feedback, the study aimed to enhance children’s learning motivation and their willingness for sustained engagement.

The study features 2 computer games (Oral Hygiene Battle and Little Monster Adventure). In the “Oral Hygiene Battle” game, the bacteria are designed as diverse and whimsical characters to guide children through the “defeating bacteria” gameplay trajectory. This process facilitates the incremental establishment of awareness regarding the importance of oral health. By performing simulated brushing maneuvers, children not only cultivate healthy oral hygiene habits but also enhance their hand-eye coordination, concentration, and gross motor development within the gaming process. The “Little Monster Adventure” game integrates characters including monsters, stars, fire, stones, and adventure-based level elements, along with auditory prompts, to create a vivid and engaging gaming environment. The game is designed to train children’s motor skills, reaction speed, and sensory integration through tasks involving limb movements, jumping, and vocalization, thereby promoting motor development and the learning of sensory integration.

Both computer games use the sensing capabilities of Scratch as the evaluative framework for assessing children’s motor actions. By integrating video sensing with sprite distance detection, the system responds to physical interactions, such as touching, jumping, and vocalization exhibited by the children during gameplay. These inputs elicit corresponding interactive responses from the game characters, including disappearing, spatial movement, and the increment or decrement of scores. Furthermore, a diverse array of auditory feedback mechanisms is integrated into the games, with specific sound effects triggered by scoring events, errors, and the conclusion of the session. These elements are designed to augment the overall playfulness and engagement of the learning experience. The researchers recorded the children’s weekly game scores on a game performance record sheet.

#### Study Procedure

Prior to the formal study, a pilot test was conducted on the self-developed computer games. The pilot participants consisted of children in the senior class (aged 4‐6) of a preschool. The researchers allowed the children to trial the games to evaluate the fluidity of the operational process. The pilot results indicated that, overall, the children could operate the games smoothly and comprehend the game rules and mechanics. However, some younger participants required assistance during their initial interactions with the games. Additionally, it was observed that sound detection within the games was susceptible to environmental factors; consequently, the game’s sensitivity parameters were adjusted to optimize performance.

The implementation process of this study was divided into 3 distinct phases: “pretest preparation,” “instructional intervention,” and “posttest assessment.” During the pretest preparation phase, conducted 1 week prior to the intervention, the researchers performed baseline assessments of the children’s “physical motor development” and “social interaction performance” across both groups. This established a baseline dataset to serve as a comparative foundation for subsequent efficacy analyses. Subsequently, the experimental group engaged in the computer game–based instructional intervention. Each instructional session followed a 3-stage pedagogical framework: motivation, developmental activity, and concluding activity. In the motivation stage, teachers used picture book stories and finger rhymes to stimulate interest. During the developmental activity stage, children were organized into groups for hands-on gameplay. They interacted with the digital scenarios through physical movements such as waving their limbs, jumping, and vocalizing. The system provided real-time audiovisual feedback, enabling the children to adjust and refine their movements accordingly. In the concluding activity stage, following the gameplay, teachers guided the children in completing worksheets and sharing their reflections to internalize the gaming experiences into concrete learning outcomes. In contrast, the control group did not receive the computer game instruction and maintained the preschool’s standard curriculum and daily routines. Upon completion of the 4-week program, all participants underwent posttest assessments regarding their physical motor and social interaction development. Through observation records and descriptive documentation of the children’s learning status, the study collected performance data for all participants before and after the computer game intervention.

### Data Analysis

Statistical analysis was performed using SPSS Statistics (version 20.0; IBM Corporation) software. Descriptive statistics were used to analyze the children’s demographic variables. Independent samples 2-tailed *t* tests were conducted to compare the differences between the experimental and control groups regarding age, body height, weight, and BMI. To evaluate the effectiveness of interventions on the physical and social development of preschool children, we used an analysis of covariance with previous test scores as a covariate to adjust for baseline differences. Independent samples 2-tailed *t* tests were conducted to compare the differences between the experimental and control groups regarding physical motor and social interaction development in the pretest. Furthermore, paired samples 2-tailed *t* tests were used to examine the differences in these developmental domains within each group before and after the computer game intervention. In addition to quantitative analysis, the observation records were presented in a descriptive manner to document interactive behaviors and emotional states during the instructional intervention. Statistical results were considered statistically significant with *P*<.05.

### Ethical Considerations

This study was conducted in strict accordance with the ethical principles for research involving human participants. The research protocol was reviewed and formally approved by the Central Regional Research Ethics Committee of China Medical University, Taichung, Taiwan (CRREC-114016). Prior to data collection, written informed consent was obtained from the legal guardians of all participating children. Furthermore, verbal assent was sought from and granted by the children themselves, ensuring their voluntary participation. To safeguard participant privacy and confidentiality, all collected data were fully anonymized and deidentified; no personally identifiable information was stored or used during the analysis. Regarding participant compensation, no financial incentives were provided. Finally, the authors certify that no individual participants can be identified in any images, figures, or supplementary materials included in this paper, as all visual data have been processed to ensure complete anonymity.

## Results

### Participant Demographics

**Figure 1.**
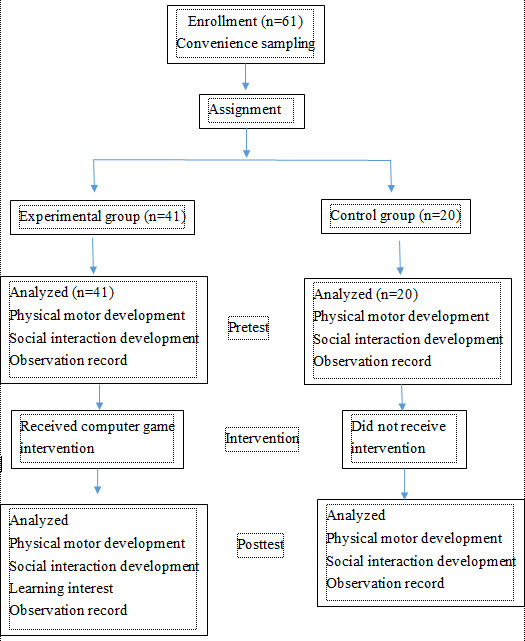
Flow of participants through each stage of the study.

The demographic characteristics of the participants are summarized in Table 1. This study enrolled a total of 61 young children, with 41 assigned to the experimental group and 20 to the control group. The flow of participants through each stage of the experiment—enrollment, assignment, follow-up, and analysis—is detailed in Figure 1. The mean age of the entire sample was 5.12 (SD 0.62) years. No statistically significant differences were observed between the experimental and control groups across several variables, including age, height, weight, BMI, gender, birth order, and parental education level. These findings indicate that the 2 groups were homogeneous regarding their background characteristics, ensuring comparability between the groups.

**Table 1.** Demographics of participants.

Variables	All (N=61)	Experimental group (n=41)	Control group (n=20)	*t* test (*df*) and χ^2^ (*df*)	*P* value
Age (y), mean (SD)	5.12 (0.62)	5.10 (0.61)	5.16 (0.66)	−0.35^a^ (59)	.73
Body height (cm), mean (SD)	106.31 (6.03)	106.22 (6.65)	106.50 (4.63)	−0.17^a^ (59)	.87
Body weight (kg), mean (SD)	17.72 (2.45)	17.54 (2.63)	18.10 (2.05)	−0.84^a^ (59)	.40
BMI, mean (SD)	15.67 (1.80)	15.54 (1.98)	15.93 (1.37)	−0.79^a^ (59)	.44
Gender, n (%)				0.20^b^ (1)	.66
Man	36 (59.0)	25 (60.9)	11 (55)		
Woman	25 (40.9)	16 (39.0)	9 (45)		
Birth order, n (%)				1.40^b^ (2)	.50
First	30 (49.2)	18 (43.9)	12 (60)		
Second	27 (44.3)	20 (48.8)	7 (35)		
Third or above	4 (6.6)	3 (7.3)	1 (5)		
Father’s education level, n (%)				2.03^b^ (3)	.57
High school or below	11 (18.0)	6 (14.6)	5 (25)		
College	47 (77.0)	33 (80.5)	14 (70)		
Graduate school above	3 (4.9)	2 (4.9)	1 (5)		
Mother’s education level, n (%)				2.51^b^ (2)	.29
High school or below	9 (14.8)	4 (9.8)	5 (25)		
College	48 (78.7)	34 (82.9)	14 (70)		
Graduate school above	4 (6.6)	3 (7.3)	1 (5)		

a Statistical values were calculated using an independent samples 2-tailed *t* test.

b Statistical values were calculated using the *χ*2 test.

### Performance in Computer Games

Table 2 presents the scores for the experimental group. In the “Oral Hygiene Battle” game, scores increased progressively from week 1 to week 4, with week 4 scores being significantly higher than week 1 scores. For the “Little Monster Adventure” game, although a slight upward trend was observed from week 1 to week 4, the difference did not reach statistical significance.

**Table 2.** Performance status of computer game scores.

Week	Oral Health Defense Battle	Little Monster Adventure
Week 1, mean (SD)	69 (32)	16 (3)
Week 2, mean (SD)	94 (25)	17 (4)
Week 3, mean (SD)	107 (20)	17 (3)
Week 4, mean (SD)	109 (20)	17 (2)
2-tailed *t* test (*df*)	−9.27 (59)	−1.45 (59)
*P* value	<.001^a^	.16^a^

a Week 1 vs week 4.

### Physical Motor Development

As shown in Table 3, the results revealed a significant main effect of the group on the physical development total score after adjusting for pretest values (*F*_1,58_=8.15, *P*<.001). The adjusted posttest mean for the experimental group was significantly higher than that of the control group, indicating a robust overall improvement in motor skills following the intervention. From further item-level analysis, the results indicate that after adjusting for baseline scores, the experimental group demonstrated significant improvements over the control group in several advanced motor skills, including standing on one leg for at least 5 seconds, walking forward 5 steps alternating heel-to-toe, and hopping on one leg at least 5 times. Conversely, no significant differences were observed between the groups for more fundamental tasks, such as walking in a straight line, standing on one leg for 3 seconds, jumping on one leg twice without support, and hopping 4 steps on one leg (Table 3).

Prior to the intervention, an independent samples 2-tailed *t* test was conducted to ensure baseline equivalence. There were no significant differences between the experimental group and the control group in any of the physical motor development items at the pretest.

Paired samples 2-tailed *t* test analysis was used to analyze changes in children’s physical motor development before and after the intervention. The experimental group showed significant improvements in all physical motor development indicators after the intervention. In contrast, the control group showed statistical significance in only 4 indicators: standing on one leg for 3 seconds (*t*_59_=−2.18, *P*=.04), continuous jumping on one leg (2x*; t*_59_=−3.20, *P*<.001), hopping 4 steps on one leg (*t*_59_=−3.20, *P*<.001), and walking forward heel-to-toe (*t*_59_=-2.517, *P*=.02) after the intervention.

**Table 3.** The physical development of preschool children between the experimental and control groups^a^.

Variables and items	Experimental group (n=41), mean (SD; 95% CI)	Control group (n=20), mean (SD; 95% CI)	*F* test (*df*)	*P* value	η_p_^2^
Physical development			8.15 (1, 58)	<.001^b^	0.12
Pretest	4.17 (0.36; 4.06-4.28)	3.91 (0.64; 3.63-4.19)			
Posttest	4.32 (0.33; 4.22-4.42)	4.03 (0.68; 3.73-4.33)			
Adjusted posttest	4.33 (0.06; 4.21-4.45)	4.02 (0.09; 3.84-4.20)			
Individual items					
1. Can go up/down stairs independently			3.17 (1, 58)	.08	0.05
Pretest	3.63 (0.70; 3.42-3.84)	3.65 (0.67; 3.36-3.94)			
Posttest	3.95 (0.22; 3.88-4.02)	3.80 (0.52; 3.57-4.03)			
Adjusted posttest	3.95 (0.05; 3.85-4.05)	3.80 (0.07; 3.66-3.94)			
2. Can walk in a straight line			1.22 (1, 58)	.27	0.02
Pretest	3.66 (0.53; 3.50-3.82)	3.70 (0.66; 3.41-3.99)			
Posttest	3.88 (0.33; 3.78-3.98)	3.80 (0.62; 3.53-4.07)			
Adjusted posttest	3.88 (0.05; 3.78-3.98)	3.79 (0.07; 3.65-3.93)			
3. Can stand on one leg (3 s)			0.63 (1, 58)	.43	0.01
Pretest	3.46 (0.71; 3.24-3.68)	3.60 (0.82; 3.24-3.96)			
Posttest	3.83 (0.38; 3.71-3.95)	3.80 (0.62; 3.53-4.07)			
Adjusted posttest	3.85 (0.06; 3.73-3.97)	3.76 (0.08; 3.60-3.92)			
4. Can jump on one leg (2x)			0.45 (1, 58)	.50	0.01
Pretest	3.41 (0.87; 3.14-3.68)	3.45 (0.95; 3.03-3.87)			
Posttest	3.85 (0.36; 3.74-3.96)	3.80 (0.62; 3.53-4.07)			
Adjusted posttest	3.86 (0.06; 3.74-3.98)	3.79 (0.08; 3.63-3.95)			
5. Can hop 4 steps on one leg			0.93 (1, 58)	.34	0.02
Pretest	3.17 (0.95; 2.88-3.46)	3.40 (0.94; 2.99-3.81)			
Posttest	3.78 (0.48; 3.63-3.93)	3.75 (0.64; 3.47-4.03)			
Adjusted posttest	3.81 (0.07; 3.67-3.95)	3.70 (0.09; 3.52-3.88)			
6. Can stand on one leg (5 s)			20.80 (1, 58)	<.001^c^	0.26
Pretest	2.90 (0.86; 2.64-3.16)	3.10 (0.79; 2.75-3.45)			
Posttest	3.76 (0.54; 3.59-3.93)	3.20 (0.62; 2.93-3.47)			
Adjusted posttest	3.78 (0.08; 3.62-3.94)	3.16 (0.11; 2.94-3.38)			
7. Can walk forward heel-to-toe			6.83 (1, 58)	.01^d^	0.11
Pretest	3.51 (0.71; 3.29-3.73)	3.35 (0.93; 2.94-3.76)			
Posttest	3.85 (0.36; 3.74-3.96)	3.45 (0.95; 3.03-3.87)			
Adjusted posttest	3.82 (0.07; 3.68-3.96)	3.51 (0.10; 3.31-3.71)			
8. Can hop at least 5 times			85.87 (1, 58)	<.001^c^	0.60
Pretest	2.54 (0.75; 2.31-2.77)	2.80 (0.89; 2.41-3.19)			
Posttest	3.85 (0.36; 3.74-3.96)	2.85 (0.75; 2.52-3.18)			
Adjusted posttest	3.88 (0.07; 3.74-4.02)	2.79 (0.10; 2.59-2.99)			

a Pretest scores were used as the covariate. Adjusted means are estimated marginal means from the analysis of covariance model.

b *P*<.01.

c *P*<.001.

d *P*<.05.

### Social Interaction Development

The social interaction development status of the preschool children is shown in Table 4. There is no statistically significant difference between the experimental and control groups in the total social interaction score following the intervention. Similarly, no significant group effects were observed across any of the individual items. In summary, while the total score indicated a small-to-medium effect size trend, the intervention did not lead to a statistically significant enhancement in social interaction development within the study time frame.

Prior to the intervention, an independent samples 2-tailed *t* test was conducted to ensure baseline equivalence. There were no significant differences between the experimental group and the control group in any of the social interaction items at the pretest (*P*>.05).

Paired samples 2-tailed *t* test analysis was used to examine changes in children’s social interaction before and after the intervention. The children in the experimental group showed a statistically significant improvement in their ability to maintain stable and sustained attention for more than 10 minutes (*t*_59_=−3.35, *P*=.002), whereas the control group exhibited no statistically significant differences across any of the assessed dimensions of social interaction.

**Table 4.** The social interactions of preschool children between the experimental and control groups^a^.

Variables and items	Experimental group (n=41), mean (SD; 95% CI)	Control group (n=20), mean (SD; 95% CI)	*F* test (*df*)	*P* value	η_p_^2^
Social interaction			3.40 (1, 58)	.07	0.06
Pretest	3.59 (0.34; 3.49-3.69)	3.67 (0.22; 3.57-3.77)			
Posttest	3.66 (0.29; 3.57-3.75)	3.79 (0.20; 3.70-3.88)			
Adjusted posttest	3.66 (0.04; 3.58-3.74)	3.79 (0.06; 3.67-3.91)			
Individual items					
1. Can follow game rules			0.03 (1, 58)	.86	0
Pretest	3.80 (0.46; 3.66-3.94)	4.00 (0; 4.00-4.00)			
Posttest	3.85 (0.42; 3.72-3.98)	4.00 (0; 4.00-4.00)			
Adjusted posttest	3.90 (0.04; 3.82-3.98)	3.91 (0.05; 3.81-4.01)			
2. Plays games with variety			0.31 (1, 58)	.58	0.01
Pretest	3.34 (0.73; 3.12-3.56)	3.50 (0.61; 3.23-3.77)			
Posttest	3.41 (0.71; 3.19-3.63)	3.60 (0.60; 3.34-3.86)			
Adjusted posttest	3.45 (0.07; 3.31-3.59)	3.52 (0.10; 3.32-3.72)			
3. Lack of patience			0.10 (1, 58)	.75	0
Pretest	3.00 (0.55; 2.83-3.17)	3.30 (0.57; 3.05-3.55)			
Posttest	3.15 (0.65; 2.95-3.35)	3.40 (0.60; 3.14-3.66)			
Adjusted posttest	3.21 (0.08; 3.05-3.37)	3.26 (0.12; 3.02-3.50)			
4. Attention span >10 minutes			0.04 (1, 58)	.85	0
Pretest	3.39 (0.63; 3.19-3.59)	3.70 (0.66; 3.41-3.99)			
Posttest	3.61 (0.54; 3.44-3.78)	3.80 (0.52; 3.57-4.03)			
Adjusted posttest	3.68 (0.05; 3.58-3.78)	3.66 (0.07; 3.52-3.80)			
5. Maintains eye contact			0.23 (1, 58)	.63	0
Pretest	3.85 (0.36; 3.74-3.96)	3.90 (0.31; 3.76-4.04)			
Posttest	3.98 (0.16; 3.93-4.03)	3.95 (0.22; 3.85-4.05)			
Adjusted posttest	3.98 (0.03; 3.92-4.04)	3.95 (0.04; 3.87-4.03)			

a Pretest scores were used as the covariate. Adjusted means are estimated marginal means from the analysis of covariance model.

### Learning Interest in Computer Games

The preschool children’s interest in learning through computer games was assessed after the intervention. The total experimental group showed a high overall preference (mean 4.18, SD 0.79). Specifically, the “Oral Health Defense Battle” received a mean score of 4.12 (SD 1.25), and “Little Monster Adventure” received a mean score of 4.20 (SD 0.98).

### Observations of Children With Developmental Delays

Qualitative observations were recorded for 3 children with developmental delays in the experimental group. Following the intervention, changes were observed in their motor and social behaviors. Motor actions transitioned from repetitive movements to coordinated sequences. In social contexts, participants moved from passive observation to active engagement in gameplay. They also maintained sustained attention and displayed positive affect (eg, smiling and verbal excitement) during the activities.

## Discussion

### Overview

The objective of this study was to evaluate the impact of a computer game–based intervention on the motor and social development of preschool children. Our findings provide nuanced support for the research hypotheses established at the outset of the study. Regarding hypothesis 1, the results partially support the prediction that the intervention would enhance gross motor skills; specifically, children exhibited significant longitudinal improvement in advanced motor tasks such as hopping and dynamic balance, although basic tasks showed a ceiling effect. In relation to hypothesis 2, children’s strong preference for games offering immediate feedback and a high sense of achievement suggests that the digital intervention effectively bolstered intrinsic learning motivation and engagement. However, hypothesis 3 was not supported, as no significant changes were observed in social interaction indicators. This lack of significance may be attributed to the relatively short intervention duration and the high baseline scores observed among participants. These outcomes, derived from our quasi-experimental analysis, are discussed in detail below.

### Principal Findings

Regarding the game performance results of the present study, the significant improvement observed in “Oral Hygiene Defense,” contrasted with the stability in “Little Monster Adventure,” provides critical interpretations regarding game design in early childhood. The discrepancy suggests that ease of use and the level of challenge are pivotal in determining engagement. Our qualitative observations indicate that young children favor mechanics with intuitive operations and attainable high scores, which foster a sense of mastery. These results align with previous findings, suggesting that serious games enhance learning interest most effectively when they provide immediate, positive feedback [25]. The implication here is that for educational software to be successful in a preschool context, developers must balance cognitive load with contextualized, rewarding interactions to sustain intrinsic motivation.

In terms of physical development, this study showed the intervention’s impact was most prominent in advanced motor tasks (eg, hopping and heel-to-toe walking), whereas fundamental tasks reached a ceiling effect. This suggests that while basic motor functions may develop through standard curricula, targeted digital interventions are essential for achieving complex developmental milestones. Comparisons with other studies [5,26] further support the notion that “cognitive-motor” integration is highly beneficial during critical growth periods. Crucially, these findings challenge the sedentary stereotype of computer games [5,26]. Our study implies that, when appropriately designed, digital play can serve as a potent tool to stimulate sensorimotor functions rather than hindering them, providing a new perspective for “active” screen time in early education.

In contrast, the lack of statistically significant gains in social interaction (eg, rule-following and eye contact) may be attributed to the individual-centric nature of the digital platforms used, which prioritized performance over interpersonal exchange [3]. Furthermore, the high pretest means indicated a ceiling effect, suggesting that the participants already possessed strong social foundations. The study indicated that social behaviors and personality traits are typically more resistant to change and require more sustained environmental support compared to motor skills [3,17]. The implication for future research is that digital interventions at social development may require a longer duration and more collaborative, multiplayer design features to yield measurable improvements.

Finally, the high learning interest scores confirm that ease of use and a sense of accomplishment are the main drivers of children’s engagement in learning. The vibrant visuals and intuitive controls allowed children to achieve success easily, thereby stimulating their enthusiasm for learning [23]. This consistent positive reinforcement supports sustained task persistence and cultivates a more conducive learning environment relative to conventional pedagogical approaches [27]. Our study implies that integrating well-designed digital tools into preschool settings can significantly boost interest, provided that the tools are aligned with the developmental readiness of the child.

### Comparison With Prior Work

Recent research has explored the impact of computer games as a pedagogical tool on children’s fundamental motor skills and social interactions. Results indicate that digital game–based learning can enhance fundamental movement skills while fostering cooperative behaviors and emotional regulation [13,28]. These findings demonstrate that, compared with traditional training methods, computer games generally elicit higher levels of engagement and yield more positive outcomes among children. However, the majority of existing studies focus on general children, often overlooking the specific development of those with special needs or developmental delays [3,7,8]. The findings of this study corroborate the benefits of such interventions, revealing that computer game activities had a positive influence on the emotional states and participation interest of several children with special needs within the classroom. Furthermore, this study observed a significant increase in children’s sustained attention. By conducting a quasi-experiment in a Taiwanese preschool, this research provides valuable regional data that reflects local educational culture, which may diverge from the Western-centric data prevalent in mainstream journals. Regarding social interaction, this study focuses on the stability of social interaction and the nature of interest-driven engagement, establishing a more direct correlation between learning interest and social performance.

### Study Limitations

Despite the preliminary efficacy demonstrated in physical motor skills in this study, several limitations remain to be addressed. First, the relatively small sample size and the uneven distribution of participants between the experimental and control groups may have affected the statistical power. Additionally, although the duration of the intervention was sufficient to observe significant changes in motor skills, it may have been inadequate for capturing more profound or long-term transformations in social behavior. Future studies should consider a more balanced group distribution and a longer intervention period to better capture the longitudinal development of social-emotional skills in early childhood.

This study empirically validates the application potential of computer games in early childhood education. Contrary to the prevailing stereotype that electronic devices exert only negative impacts, our findings demonstrate that carefully screened and well-designed educational computer games can serve as effective auxiliary tools for promoting gross motor development. Future research could extend the intervention period to 1 semester or longer to explore the longitudinal impact of computer games on children’s prosocial behavior. Notably, this study observed that 3 children with developmental delays showed multifaceted improvements in motor coordination, social understanding, and emotional stability following the interactive computer game intervention. These observations are consistent with existing literature suggesting that the educational use of computer games has positive effects on children with autism and intellectual disabilities, highlighting significant application potential [19,23]. Consequently, future studies could expand their scope to inclusive education settings to further investigate the value of these tools in adaptive instruction.

### Conclusions

The findings of this research demonstrate that educational computer games significantly facilitate the physical motor development of preschool children while concurrently augmenting their intrinsic learning motivation. However, the results indicate no statistically significant impact on social interaction. Educational computer games have evolved beyond rudimentary pedagogical instruments, becoming pivotal catalysts for early childhood development. The significance of this study resides in its empirical validation of integrating “serious games” into formal early childhood curricula, effectively transitioning them from peripheral teaching aids to fundamental pillars of holistic development. To maximize the efficacy of these digital tools, this study advocates for interdisciplinary collaboration between pedagogical experts and software architects. Such a partnership ensures a strategic equilibrium between narrative engagement and interface usability, ultimately positioning educational computer games as essential components in fostering comprehensive child development.
